# The Biomarker Potential of Caveolin-1 in Penile Cancer

**DOI:** 10.3389/fonc.2021.606122

**Published:** 2021-03-31

**Authors:** Andrej Panic, Henning Reis, Alina Wittka, Christopher Darr, Boris Hadaschik, Verena Jendrossek, Diana Klein

**Affiliations:** ^1^Department of Urology, West German Cancer Center, University of Duisburg-Essen, University Hospital Essen, Essen, Germany; ^2^Institute of Pathology, West German Cancer Center, University of Duisburg-Essen, University Hospital Essen, Essen, Germany; ^3^Institute of Cell Biology (Cancer Research), University of Duisburg-Essen, University Hospital, Essen, Germany

**Keywords:** caveolin-1, tumor stroma, penile cancer, microenvironment, biomarker, p16, p53

## Abstract

Various types of human cancers were characterized by an altered expression of epithelial or stromal caveolin-1 (CAV1). However, the clinical significance of CAV1 expression in penile cancer remains largely unknown. Here the expression patterns of CAV1 were analyzed in a retrospective cohort (n=43) of penile squamous cell carcinomas (SCC). Upon penile cancer progression, significantly increased CAV1-levels were determined within the malignant epithelium, whereas within the tumor stroma, namely the fibroblastic tumor compartment harboring activated and/or cancer associated fibroblasts, CAV1 levels significantly decline. Concerning the clinicopathological significance of CAV1 expression in penile cancer as well as respective epithelial-stromal CAV1 distributions, high expression within the tumor cells as well as low expression of CAV1 within the stromal compartment were correlated with decreased overall survival of penile cancer patients. Herein, CAV1 expressions and distributions at advanced penile cancer stages were independent of the immunohistochemically proven tumor protein p53 status. In contrast, less differentiated p16-positive tumor epithelia (indicative for human papilloma virus infection) were characterized by significantly decreased CAV1 levels. Conclusively, we provide further and new evidence that the characteristic shift in stromal‐epithelial CAV1 being functionally relevant to tumor progression even occurs in penile SCC.

## Introduction

Penile cancer is a rare disease with an age-standardized incidence of 0.8 per 100,000 males (36,068 cases) and mortality of 0.3 (13,211 deaths) worldwide in 2020 (1, 2). Several risk factors for penile cancer have been identified, including phimosis, chronic penile inflammation (balanoposthitis related to phimosis), balanitis xerotica obliterans (lichen sclerosus), sporalene and UVA phototherapy for various dermatological conditions such as psoriasis, smoking, human papilloma virus infection (HPV) infection, rural areas, low socioeconomic status, multiple sexual partners, and early age of first intercourse (3–5). Especially HPV is an important risk factor; up to 50% of all cases of penile carcinoma are seen in presence of HPV infections (6–9). However, the impact of high-risk HPV in the prognosis of penile cancer remains unclear (10, 11). Among the different types of penile cancer, squamous cell carcinoma (SCC), accounts for > 95% of cases of penile malignancies that arise from the epithelium of the glans, the foreskin (prepuce) or the shaft (12–14). The different histological SSC subtypes and the histological grades, together with other pathologically based factors, such as histological thickness, tumor site, size, as well as vascular or perineural invasion were used to identify pathological risk groups (15–18). The overall 5-year relative survival rates are >80% for localized disease states, but decreases <50% for patients with positive lymph node status (pN1–3) (19, 20). SSC histological subtypes and/or the tumor grade together with perineural and lymphatic invasion turned out to be important prognostic factors of penile cancer (5, 15, 21). However, the prognosis for patients with systemic metastasis remains poor. It is not uncommon for patient factors to delay diagnosis and initiation of treatment because the treatment is often associated with cosmetic and functional defects. As with many other cancers, the identification of prognostic factors, particularly biomarkers in penile cancer would create the opportunity to stratify patients according to risk of cancer progression and, therefore, impact on treatment decisions. To date, potential candidate biomarkers are still not rigorous enough to be routinely used in the diagnosis and management of penile malignancy (7, 21–24).

The membrane protein caveolin-1 (CAV1) gained attraction in carcinogenesis because it was shown to be overexpressed or mutated in numerous solid human tumors (25–28). As main structural component of specialized (flask-shaped) cholesterol and sphingolipids-enriched microdomains of the plasma membrane termed caveolae, CAV1 regulates multiple cell signaling pathways and thus regulating cancer-associated processes, ranging from cellular transformation, tumor growth and angiogenesis, invasion and metastasis, to multidrug resistance cells (29, 30). Upon tumor progression, CAV1 levels strongly increase in malignant epithelial cells, which was correlated with worse clinical outcomes in a couple of cancer entities, including prostate, pancreatic, and lung cancer (31–36). Concurrently, a loss of stromal CAV1, particularly affecting cancer associated fibroblasts (CAF), could be observed, which correlated with tumor progression, therapy resistance, and predicted adverse outcome, e.g. in breast and prostate cancer (25, 37–40). Therefore, CAV1 expression levels and especially stroma-epithelial distributions have strong indications to severe as a prognostic marker. However, nothing is known about the role of CAV1 in in penile tumorigenesis. The present study aimed to explore the clinicopathological significance and the respective biomarker potential of CAV1 expression levels, in both tumor cells and the stromal compartment that houses predominately the CAFs as well as vascular structures.

## Materials and Methods

### Patients and Procedures

Patients were surgically treated between 2009 and 2018 at the Department of Urology, University Hospital Essen, University of Duisburg-Essen. Tissues from penile carcinomas were obtained during surgery according to local ethical regulations. Resected tissue specimens were processed for pathological diagnostic routine in agreement with institutional standards and diagnoses were made based on current WHO criteria (22, 41–43). The study was performed according to the Declaration of Helsinki and was approved by the local ethics committee of the University Hospital Essen (Ethik-Kommission, Medizinische Fakultät der Universität Duisburg-Essen, ethical approval number: 20-9508-BO). Human tissue samples were analyzed anonymously. Overall survival (OS) was the primary endpoint of this retrospective study; 43 patients diagnosed with penile carcinoma were included. Patients were followed from the date of surgery until July 2020 with the reverse Kaplan-Meier estimate (44). Immunohistochemistry (IHC) and immunofluorescence staining was performed on formalin-fixed and paraffin-embedded (FFPE) tissue sections (4-5 μm). p53/TP53 (BP53-12; Zytomed Systems, Berlin, Germany) and p16^INK4a^ (MSK123-05; Zytomed Systems) IHC staining were carried out on a Benchmark Ultra System (Ventana Medical Systems, Tucson, AZ, USA) with antibody visualization using the OptiView DAB IHC Detection Kit (Ventana Medical Systems) according to the manufacturer’s instructions (45). Tumor specimens with strong and diffuse nuclear and cytoplasmic staining in more than 95-98% of tumor cells were considered as p16-positive. Evaluation of p53 immunoreactivity was carried out by assessing the percentage of positive tumor nuclei as previously described (46). CAV1 (N-20; sc-894; Santa Cruz Biotechnology, CA, USA) IHC was performed as previously described (39, 40, 47). In brief, samples were prepared by using a descending alcohol series and incubation with citrate buffer, pH 6.1 as target retrieval solution. Afterwards slides were blocked with a 2% fetal calf serum in phosphate buffered saline (blocking solution) to reduce unspecific interactions and primary antibody was incubated overnight at 4°C. CAV1 was detected by a horseradish-peroxidase conjugated secondary antibody and DAB-staining. Nuclei were counterstained with hematoxylin. Combined quantitative and qualitative evaluation of CAV1-immunoreactivity was performed blinded to clinical/follow-up data using a CAV1 immunoreactivity score for low (0-0.5), moderate (>0.5-1.5), and high (>1.5-2) CAV1 expression levels either estimated for the immunoreactivity of tumor cells or of the stromal (fibroblastic) compartment. The immunoreactivity score takes into account both the percentage of positive cells and staining intensity (48, 49). The proportion (and the intensity of immunoreactivity) of stained cells was scored as follows: 0-0.5, no staining or ≤10% stained cells (week staining); <0.5-1.5, 11-50% stained cells (moderate staining), and 3, 51-100% (usually >80%) stained cells (strong staining). Data analysis was performed with Prism 8 software (GraphPad, La Jolla, CA, USA). Statistical significance was set at the level of p ≤ 0.05 (*p ≤ 0.05, **p ≤ 0.01, ***p ≤ 0.005, ****, p ≤ 0.001). Differences in survival between the groups were determined with the log rank test. Cox regression was performed to assess hazard ratios (HR). Mean values of clinicopathological parameters and immunohistochemical results were calculated and used for analysis of standard error (SEM). Statistical significance was evaluated by 1-way ANOVA followed by multiple comparisons post-tests as indicated in the respective figure legend.

## Results

### Epithelial-Stromal CAV1 Distributions of CAV1 in Penile Carcinoma

**Figure 1:**
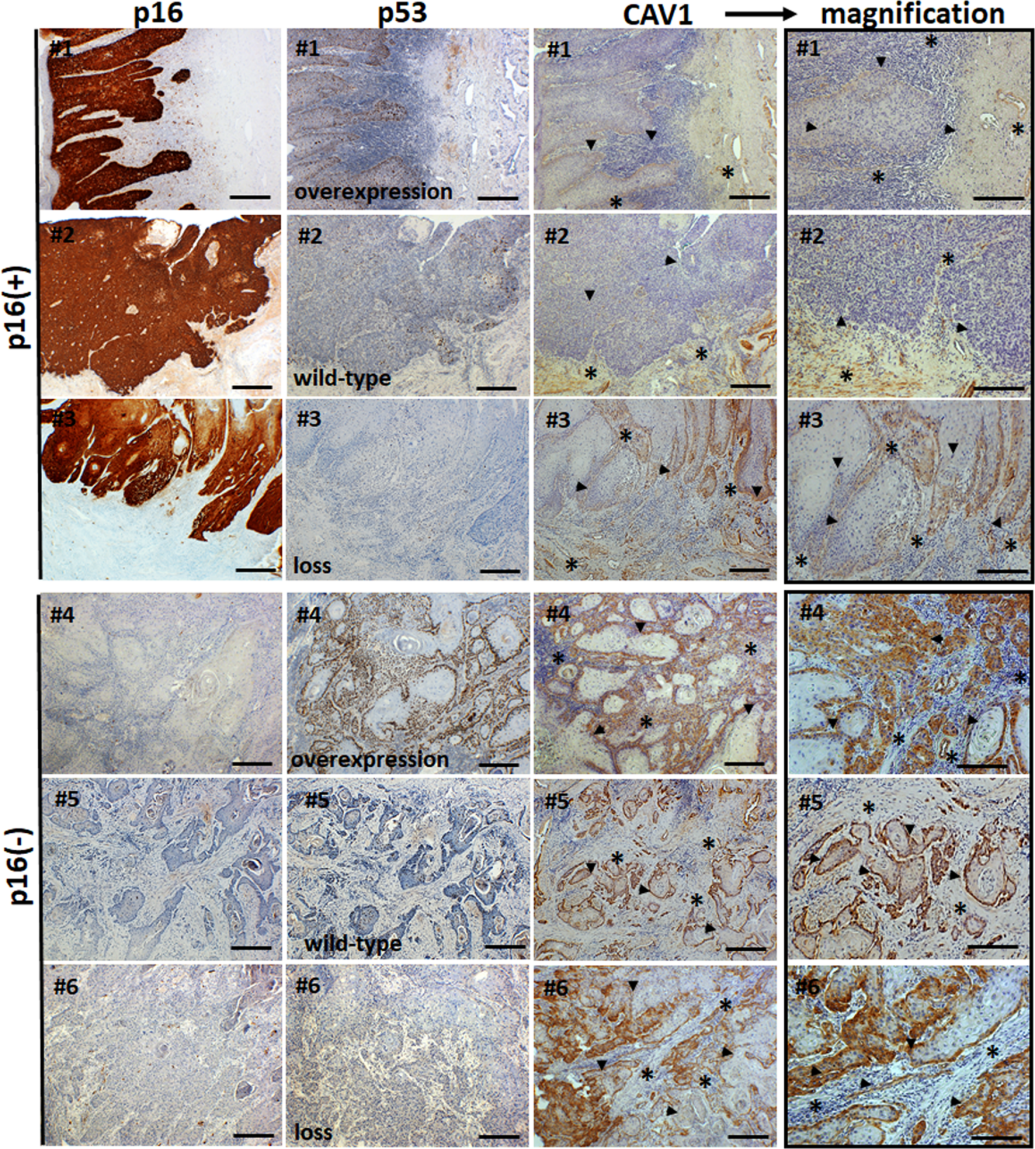
Immunohistological analysis of CAV1 levels and epithelial-stromal CAV1 distributions in correlation with p16 and p53 levels in human penile tumor tissues. Paraffin-sections of human penile carcinomas were stained for p16, p53 and CAV1 in combination with DAB (brown). Representative images of p16-positive [p16(+)] and p16-negative [p16(-)] tumor specimen are shown. The 53 status (overexpression, wild-type and loss) is indicated. Asteriks (*) mark stromal compartments and bold arrows point to epithelial structures. Sections were counterstained using hematoxylin. # indicates different patients. Scale bar: 200 µm, scale bar of higher magnification images (CAV1): 75 µm.

The clinicopathologic characteristics of the 43 penile carcinoma patients who have been surgically treated in our clinic and were retrospectively analyzed are listed in **Table 1**. CAV1 immunostaining was performed in order to investigate CAV1 expression levels as well as epithelial-stromal CAV1 distributions in penile carcinoma specimen according to the p16 (as a surrogate marker for the HPV infection) and p53 (TP53) status (**Figure 1**). CAV1 was expressed in all cancerous penile tissue in variable amounts, predominantly localized in the cell membrane and cytoplasm. In p16-positive tumors that generally displayed a less differentiated phenotype, CAV1 expression was not present or rather low in malignant epithelial cells (**Figure 1** #1-3, arrowheads). Even within the tumor stroma, CAV1 expression was hardly detectable and predominately restricted to the vascular compartment (**Figure 1**, #1-3, asterisks). Only two tumors of the p16-positive group (n=17) showed a stronger epithelial CAV1 immunoreactivity, and two (other) tumors showed an increased CAV1 immunoreactivity within the stromal compartment (not shown). Among the p16-negative tumors (n=26), tumors with low, moderate and high scores of CAV1 expressions within the tumor cells could be observed. As compared to the p16-positive tumors, CAV1 levels within the malignant epithelium were increased (**Figure 1**, #4-6 arrows). The CAV1 immunoreactivity within the stromal compartment seemed to be negatively regulated: with increasing epithelial CAV1 expressions, decreasing CAV1 expression levels within the stroma were observed (**Figure 1**, #4-6 asterisks). Furthermore, CAV1 expression levels within penile carcinoma turned out to be p53 independent, as the characteristic epithelial-tumoral CAV1 shift observed in p16-negative tumors did not correlate to the amount of p53 of respective specimen (**Figure 1**).

**Table 1:** Baseline patient demographics and histopathology (n = 43). 29 patients underwent partial amputation surgery (local excision and destruction of diseased tissue) or total penis amputation (14). The smoking status was unknown.

Characteristics	Sub-characteristics	Value (n %)
Age (range)		62,9 +/- 12,7 (39-90)
Stage (pT)	pT1	21 (48.8%)
	pT2	16 (37.2%)
	pT3	6 (14%)
Lymph node metastasis (pN)	pN0	4 (9.3%)
	pN1-N2/3	5 (11.6%)
	pNX	34 (79.1)
Distant metastasis (M)	M0	1 (2.3%)
	M1	1 (2.3%)
	MX	41 (95.3%)
Histologic grade	G1	8 (18.6%)
	G2	23 (53.5%)
	G3	9 (20.9%)
	GX	3 (7%)
Chemotherapy Other	Paclitaxel+Cis-/Carboplatin	12 (27.9) 2 (4,7%)
p16 status (IHC)	negative	26 (60.5%)
	positive	17 (39.5%)
p53 status (IHC)	loss	7 (16.3%)
	wild-type	26 (60.5%)
	overexpression	10 (32.3%)
CAV1 scores (epithelium)	low (0-0.5)	17 (39.5%)
	moderate (>0.5-1.5)	15 (34.9%)
	high (>1.5-2)	11 (25.6%)
CAV1 scores (stroma)	low (0-0.5)	12 (27.9%)
	moderate (>0.5-1.5)	13 (30.2%)
	high (>1.5-2)	18 (41.9%)
Overall Survival, days (range)	death	670 +/- 754 (40-3179)
	alive (until July 2020)	+/- 978 (523-3693)

### Clinical Significances of Epithelial-Stromal CAV1 Expression Levels

**Figure 2:**
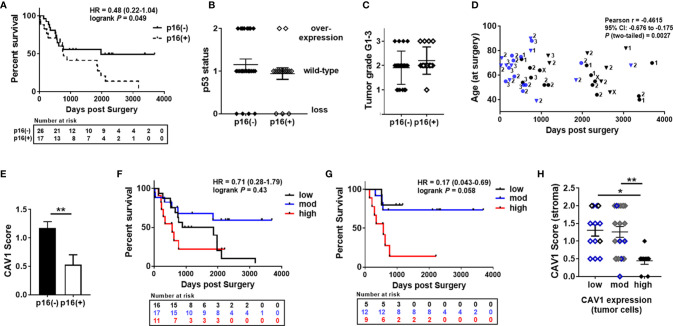
Correlation of epithelial-stromal CAV1 expressions in penile cancer as related to p16 and p53 expressions. **(A)** Overall survival curves for patients with p16 expressing (n= 17; dashed line) or p16-negative (n=26; black line) tumors. HR, hazard ratio and log-rank P values are indicated. **(B)** p16 expressing tumors were classified according to their p53 immunoreactivity (p53 status: overexpression, wild-type, loss). **(C)** p16 expression in tumors were related to their grade of differentiation: low grade (G1; n= 8), intermediate grade (G2; n=23) and high grade (G3; n=9). **(D)** Tumor grades (displayed by numbers) were further analyzed according to the respective patient age (at the time of surgery) and the overall survival time (in days) post-surgery. The blue color depicts patient’s death. p16-positivity is indicated by rectangles. Pearson correlation coefficient (Pearson r), 95% confidence interval (CI) and two-tailed P as analyzed for tumor grade versus days post-surgery were indicated (R squared = 0.213). Pearson r = -0.2205 (95%CI: -0.4885 to 0.0855; R squared = 0.049; P = 0.155) for the patients age versus days post-surgery (GX; n=3: The tumor grade could not be identified.) **(E)** p16 expressing tumors were classified according to their CAV1 immunoreactivity score for low (0-0.5), moderate (>0.5-1.5), and high (>1.5-2) CAV1 expression levels within the tumor epithelium. **P < 0.01 by unpaired t-test (two-tailed). **(F)** Overall survival curves for patients with low (n=17), moderate (n=15) and high (n=11) CAV1 expressions in tumor cells. HR, hazard ratio and log-rank P (low versus high CAV1 levels) are indicated. HR=0.36 (0.12-1.11), log-rank P (Mantel-Cox) =0.047 (moderate vs. high CAV1). **(G)** Overall survival curves for patients with p16-negative tumors and low (n=8), moderate (n= 6) and high (n=12) CAV1 content within tumor cells. HR and log-rank P (low versus high CAV1 levels) are indicated. HR=0.22 (0.058-0.80), log-rank P=0.014 (moderate versus high). **(H)** The differential CAV1 scores within the epithelial compartment were correlated to the respective CAV1 immunoreactivity scores of the tumor stroma (n=17 for low, n=15 for moderate, and n=11 for high epithelial CAV1). p16-positivity is indicated by the blue color. *P<0.05, **P<0.01 by one-way ANOVA followed by post-hoc Tukey’s multiple comparisons test.

**Figure 3:**
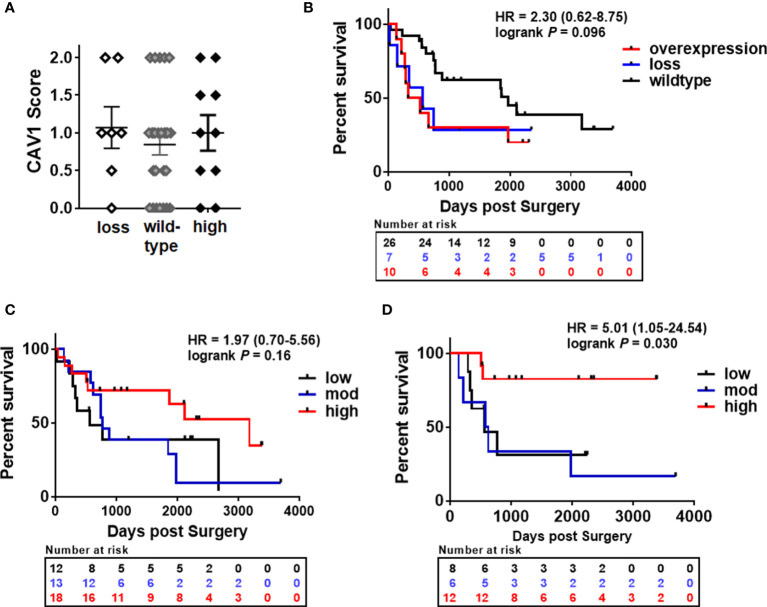
Overall survival as related to and p53 expressions and according to the expression of stromal CAV1 in penile cancer. **(A)** Differential p53 expression levels were classified according to their CAV1 immunoreactivity score for low (0-0.5), moderate (>0.5-1.5), and high (>1.5-2) CAV1 expression levels of respective tumor cells (p53 overexpression n=10, p53 wild-type n= 26, p53 low n= 7). **(B)** Overall survival curves for patients according to the p53 expression in tumors. HR and log-rank P (wild-type: blue line versus loss: black line) are indicated. HR=2.28 (0.80-6.49) log-rank P=0.053 (overexpression: red line versus loss). **(C)** Overall survival curves for patients with low (n=12), moderate (n=13) and high (n=18) CAV1 content within the tumor stroma. HR and log-rank P (low versus high CAV1 levels) are indicated. HR=2.33 (0.92-5.91), log-rank P=0.056 (moderate versus high). **(D)** Overall survival curves for patients with p16-negative tumors and low (n=8), moderate (n= 6) and high (n=12) CAV1 content within the tumor stroma. HR and log-rank P (low versus high CAV1 levels) are indicated. HR=6.22 (1.21-32.02) log-rank P=0.0025 (moderate versus high).

**Figure 4:**
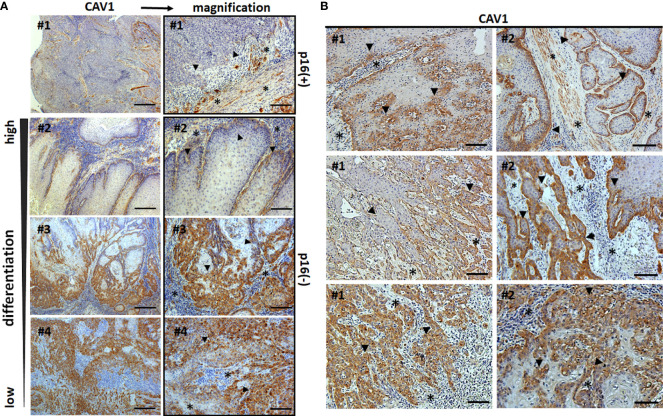
Epithelial-stromal CAV1 distributions in correlation with malignant epithelial differentiation patterns. Paraffin-sections of human penile carcinomas were stained for CAV1 and analyzed according to the tumor grade. **(A)** Representative images of well differentiated (low-grade, G1), moderately differentiated (G2) and poorly differentiated (high grade, G3) tumors are shown. P16 status is indicated. Scale bar: 200 µm, higher magnifications: 50 µm. **(B)** Representative images of different areas with variable (decreasing tumor cell) differentiation patterns within the same (p16-negative) tumor specimen are shown. Sections were counterstained using hematoxylin. Asterisks (*) mark stromal compartments and bold arrows point to epithelial structures. # indicates different patients.

**Figure 5:**
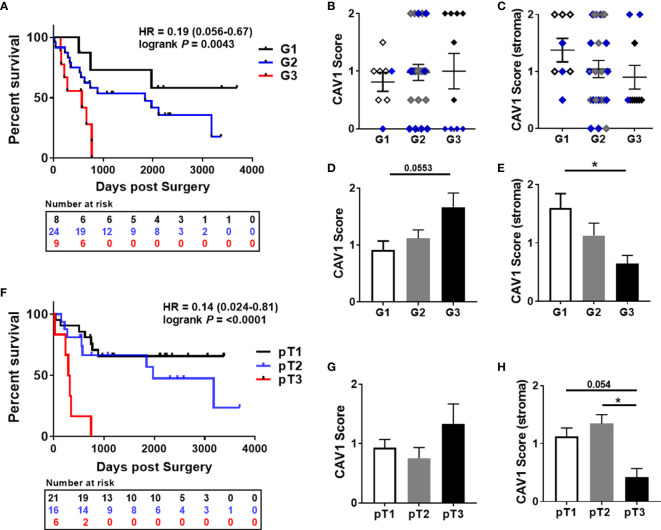
Prognostic impacts of epithelial-stromal CAV1 expressions in penile cancer in relation to the tumor grade. **(A)** Overall survival curves for penile cancer patients according to the tumor grade (low-grade/G1 n=8; moderate/G2 n=23; high grade/G3 n=9). HR and log-rank P (G1 versus G3) are indicated. HR=0.46 (0.17-1.24), log-rank P=0.20 (G1 versus G2); HR=0.35 (0.12-1.08), log-rank P = 0.010 (G2 versus G3). **(B)** According to the tumor grade, the CAV1 immunoreactivity score for low (0-0.5), moderate (>0.5-1.5), and high (>1.5-2) CAV1 expression levels of respective tumor cells was estimated. **(C)** Further on, the CAV1 immunoreactivities of the tumor stroma were determined in correlation with the tumor grades. P16-positivities are indicated by the blue color. According to the tumor grade, CAV1 scores only of the p16-negative tumor epithelium **(D)** and of the tumor stroma **(E)** were analyzed. * P<0.05 by one-way ANOVA followed by post-hoc Dunnett’s multiple comparisons test. **(F)** Overall survival curves for penile cancer patients according to the tumor stage (pT1 n=21; pT2 n=16; pT3 n=6). HR and log-rank P (pT1 versus pT3) are indicated. HR=0.23 (0.053-1.01), log-rank P=0.20 (pT2 versus pT3). CAV1 immunoreactivities of the tumor epithelium **(G)** and of the stroma(**H**) were determined in correlation with the tumor stages. *P<0.05, by one-way ANOVA followed by post-hoc Tukey’s multiple comparisons test.

To investigate the effect of epithelial-stromal CAV1 levels in correlation with p16 and p53 status on malignant progression, the correlation between CAV1 protein expression and the clinicopathologic features were examined respectively (**Figure 2**). First of all, a significant difference concerning the overall survival (OS) could be estimated for p16-positive and non-p16-related subtypes of penile SCC, with a reduced OS of patients with p16-positive penile tumors (**Figure 2A**). p53 expression levels did not correlate with the p16 status, although there was a trend of p16-negative tumors being associated with p53 overexpression (**Figure 2B**). p16-positive tumors generally tended to have a less differentiated phenotype of malignant epithelial cells (**Figure 1** #1, #2), but the there was no significant difference between the p16 status and the tumor grade (G1-G3; **Figure 2C**). The tumor grade was found to correlate with the patient age (at the time of surgery), whereas the overall survival time did not correlate with the patient age (**Figure 2D**). Of note, a significant lower CAV1 content in malignant epithelial cells as determined by significant lower CAV1 scores was present in p16 expressing penile tumors (**Figure 2E**). Moreover, CAV1 expression levels within the tumor epithelium seem to correlate with OS: with increasing CAV1 scores of the tumor epithelium the OS deteriorated (**Figure 2F**), an effect that was even more prominent when only the p16-negative tumors (n=27) were investigated (**Figure 2G**), most likely due to the fact that p16-positive tumors were shown to express less epithelial CAV1 (**Figure 2E**). A strong tumor epithelial CAV1 immunoreactivity as shown by the high CAV1 scores correlated with low CAV1 contents within the tumor stroma (**Figure 2H**). Of note, tumoral CAV1 expression levels did not correlate with the p53 status (**Figure 3A**), although loss of p53 was associated with a better OS (**Figure 3B**). The disadvantageous effect of a stromal CAV loss could be further emphasized by the fact that stromal CAV1 expressions in relation to OS revealed a better OS of respective patients with tumors harboring high stromal CAV1 levels (**Figure 3C**), particularly when considering p16-negative tumors only (**Figure 3D**). Although epithelial CAV1 levels were rather low in p16-expressing tumors, and thus in tumors with lower differentiation pattern, the impression had solidified that CAV1 was more expressed in malignant epithelial cells that bear a more undifferentiated phenotype at least in p16-negative tumors (**Figure 4**). Indeed, CAV1 expression in p16-negative tumors was predominately localized in less differentiated tumor cells with increasing epithelial CAV1 expression levels according to increasing tumor grades and thus decreasing differentiation patterns of the tumor cells (**Figure 4A**). Even within the same (p16-negative) tumor specimen, CAV1 immunoreactivity in epithelial cells was strongly increased in tumor cells with a less differentiated phenotype (**Figure 4B**). In contrast, CAV1 expression within the tumor stroma was decreasing with upon tumor progression (**Figures 4** **and** **5**). As expected, with increasing tumor grades and stages, the OS of penile cancer patients deteriorated (**Figures 5A, F**). Similarly, increasing CAV1 levels in tumor epithelial cells were accompanied by with decreasing CAV1 levels within the tumor stroma as related to increasing tumor grades (**Figures 5B, C**), an effect that was significant when considering p16-negative tumors only (**Figures 5D, E**). A loss of stromal CAV1, when epithelial CAV levels seem to increase, was further confirmed at advanced tumor stages (**Figures 5G, H**).

## Discussion

Current research efforts in penile cancer, like for many other cancers, concentrate on the identification and functional characterization of biomarkers with the potential to be modulated as valuable target for cancer therapy (7, 50–52). Patients with penile tumors that are characterized aggressive pathological features (e.g. increasing tumor grades) are associated with the highest risk for locoregional metastasis (53, 54). The occurrence of lymph node recurrence worsens tumor-specific survival levels, and patients with systemic metastasis can only expect an extremely poor prognosis (22, 55). Thus, the most important prognostic factor for survival of penile cancer patients is the complete and thorough assessment of (regional) lymph nodes spreading and penetration upon tumor progression (56). Therefore, molecular factors, particularly biomarkers were urgently needed in penile carcinogenesis that could be of relevance for therapeutic interventions in terms of targeted (therapeutic) agents for the use as first and especially as second-line treatment for patients with refractory disease (53). Here we showed now that (human) penile cancer specimen with increasing tumor grades and stages exhibited increased epithelial CAV1 levels, whereas in parallel a reduction of CAV1 in the tumor stroma (especially CAFs) was detected; and a loss of stromal CAV1 is well known to correlate with a more reactive phenotype in advanced carcinomas (25, 39, 40). This characteristic epithelial-stromal CAV1 shift was found to be functionally relevant to tumor progression and correlated with reduced overall survival. Today, there is increasing evidence that the tumor-stromal environment is not just a supporting tumor compartment but rather a key player in carcinogenesis, and impacting on cancer cell invasiveness, progression and potentially therapy resistance (57–59). Upon malignant transformation, tumor cells modulate their surrounding stroma as they grow that in turn synergistically impacts on tumor progression (60, 61). Herein, the integral membrane protein CAV1 that is abundantly expressed stromal cells (fibrocytes, fibroblasts, smooth muscle cells, adipocytes, endothelial cells), and to a variable degree in epithelial cells, gained attraction (29, 30, 62). Early in tumorigenesis CAV1 levels may decline and allow tumor cells to multiply (25, 63, 64). Upon tumor progression, an up-regulation of CAV1 could then be observed, while stromal CAV1 expression levels decline, in particular in the fibroblastic compartment; effects that were found to be functionally relevant to tumor progression, invasion, metastasis and resistance to cancer therapeutic treatment (25, 27, 65).

Invasive low-grade penile neoplasms are expected to have an excellent prognosis whereas invasive high-grade tumors (with regional or systemic dissemination) have a worse clinical outcome (19, 20, 66). Accordingly, we demonstrate that patients’ survival significantly decreased with increasing tumor grades as well as increasing tumor stages, and that higher penile tumor grades (as well as higher tumor stages) were characterized by increased CAV1 expression levels within the malignant tumor cells while there was a significant loss of stromal CAV1. Furthermore, it was already reported that HPV-positive tumors could account for a better prognosis strongly suggesting the use of the HPV status of the tumor tissues as an important prognostic marker (67, 68). This remains controversial since there were other studies reporting that the HPV status was not predictive of outcome (69). In general, p16 has been demonstrated to be an adequate surrogate for high-risk HPV, whereas the gold standard for HPV testing in tumors uses still polymerase chain reaction (7, 70). In the present retrospective cohort, p16 expression levels were significantly correlated with decreased CAV1 expression levels in penile tumor cells, which together with the decreased overall survival of CAV1-overexpressing tumors, would support the idea that p16-positive tumors could account for a better clinical outcome. CAV1 was already found to be down-regulated in cells transformed by HPV (in a p53-dependent manner) (71). The commonest HPV subtypes in penile cancer are types 16 and 18 and the risk of penile cancer is increased in patients with condyloma acuminate (72, 73). In general, the HPV types 16 and 18 harbor the potential to induce tumorigenesis (74, 75). The HPV early genes E6 and E7 are known to disrupt cell cycle regulations by inactivating two tumor suppressors, the retinoblastoma protein (*RB*) and the *p53* transcription factor. Interestingly, restoration of CAV1 expression was able to suppress this HPV-mediated malignant transformation (71, 76). For penile cancers lacking HPV infection, p53 mutations were thought to foster tumorigenesis (76–78). Deletions or mutations of were shown to mediate cell cycle progression, malignant growth of primary penile carcinomas, and correlated negatively with cancer-specific survival (79). Although we did not investigated functionality of the p53 protein in our cohort, a slight trend for increasing p53 levels in p16-negative tumors was observed, and higher p53 expression levels were associated with a poorer overall survival.

In support of clinical utility in penile cancer, further potential biomarkers have been studied, including squamous cell carcinoma antigen, C-reactive protein as well as proliferation markers (like proliferating cell nuclear antigen, cyclin D1 or Ki-67), unfortunately with only minor degree of evidence (7, 52). Therefore, the impact of these data on personalized strategies for an optimized cancer therapy remains unclear. One example for targeted therapeutic agents in penile carcinoma addressed growth factor receptors with tyrosine kinase activity, particularly the anti-epidermal growth factor receptor (EGFR) by the use of respective monoclonal antibodies (50, 51). EGFR overexpression turned out to be a common feature of penile carcinomas, independently of histologic grade or subtype, and HPV presence (80). However, when used as second-line treatment for patients with refractory disease only modest results were achieved (81). Similar the EGFR-dependent activation of the Ras/Raf/mitogen-activated protein kinase-extracellular signal-regulated kinase-signaling pathway, and as shown for multiple other cancers, activation of the phosphatidylinositol-3-kinase (PI3K), protein kinase B (PKB/AKT), and mammalian target of rapamycin (mTOR) pathway is a frequent event in carcinogenesis that facilitates tumor formation, progression and therapy resistance (82–84). Herein, a high frequency of PIK3CA (the catalytic subunit alpha of PI3K) copy number gains were reported as primary method for the activation of the PI3K-AKT-mTOR pathway in penile carcinogenesis (82, 83, 85). CAV1 was found to activate AKT in prostate cancer, and potentially other malignancies, finally leading to the increased phosphorylation of multiple AKT substrates that mediated increased cancer cell invasiveness (86, 87). Own studies further confirmed that CAV1-dependent AKT signaling, among others, is an important factor for modulating tumor as well as stromal cells proliferation and survival upon cancer therapy (25, 39, 40, 47). Thus, further investigation of epithelial-stromal CAV1 functions as well as the identification of decisive CAV1 downstream targets may allow to characterize and in turn modulate the sensitivity of epithelial tumors to cancer therapy. Unfortunately, the main limitation concerning penile cancer is based on the small number of available patients, which makes prospective randomized studies in a certain region impossible (16, 22, 88). Most of the available data come from small retrospective studies, as investigated here in a small retrospective cohort of 43 penile SCC. At the same time, no reports considering the role of CAV1 in penile tumors were available. Thus, international collaborations are needed to collect data and gain knowledge on penile SCC prior enabling potential clinical trials (16). Additional available patients would even allow us to evaluate the impact of the characteristic shift in stromal‐epithelial CAV1 according to the histologic subtype in penile SCC. Penile SCC can broadly be divided into usual SCC, verruciform tumors, basaloid carcinomas (80). Whereas the prognosis of usual SCC largely depends on location, stage and grade, verruciform tumors were found to have a good prognosis, while basaloid carcinomas were associated with a poor prognosis and frequently early inguinal nodal metastasis (89–91). Together with the fact that certain cell growth and transformation factors (e.g. the insulin-like growth factor-1 receptor) could already be significantly associated with histologic subtype (and grade), and thus being indicative for prognosis (92), a differential CAV1 distribution according to the histologic subtype might have prognostic relevance and that modulating CAV1 could be useful in treating patients with penile SCC.

## Conclusions

A characteristic shift in epithelial-stromal CAV1, as known for many other cancer entities during cancer development and progression, was established here for penile SCC. Increasing CAV1 levels in the penile tumor cells of advanced tumor grades and stages were accompanied by a loss of CAV1 within the tumor stroma, a finding that showed a significant correlation with clinicopathological features of penile SCC, particularly correlating with a reduced overall survival. As known from other cancer entities, epithelial-stromal CAV1 expression levels have the potential to serve as novel biomarker to monitor cancer progression and even therapy resistance. Conformingly, we provide further and new evidence that the characteristic shift in stromal‐epithelial CAV1 being functionally relevant to tumor progression even occurs in penile SCC. Larger cohorts of patients as well as respective functional studies are highly desired to proof the biomarker potential of CAV1 definitely, as well as to identify the underlying biological mechanisms.

## Data Availability Statement

The raw data supporting the conclusions of this article will be made available by the authors, without undue reservation.

## Ethics Statement

The studies involving human participants were reviewed and approved by the local ethics committee of the University Hospital Essen (Ethik-Kommission, Medizinische Fakultät der Universität Duisburg-Essen, ethical approval number: 20-9508-BO). The patients/participants provided their written informed consent to participate in this study.

## Author Contributions

AP: conception and design, collection and/or assembly of data, data analysis and interpretation, manuscript writing, and final approval of manuscript. HR: collection and/or assembly of data, provision of study material or patients, and final approval of manuscript. AW: collection and/or assembly of data and final approval of manuscript. CD: collection and/or assembly of data and final approval of manuscript. BH: administrative support, financial support, and final approval of manuscript. VJ: administrative support, financial support, and final approval of manuscript. DK: conception and design, collection and/or assembly of data, data analysis and interpretation, manuscript writing, financial support, and final approval of manuscript. All authors contributed to the article and approved the submitted version.

## Funding

This work was supported by grants of the DFG (GRK1739 1/2).

## Conflict of Interest

BH reports personal fees from ABX, Bayer, Lightpoint Medical, Inc., Janssen R&D, Bristol-Myers-Squibb, and Astellas, and travel from AstraZeneca, Janssen R&D, and Astellas.

The remaining authors declare that the research was conducted in the absence of any commercial or financial relationships that could be construed as a potential conflict of interest.
